# How Thermal Patterns Change During Dehydration in Non‐Vascular Epiphytic Communities

**DOI:** 10.1002/ece3.71756

**Published:** 2025-07-10

**Authors:** Giulia Canali, Pilar Hurtado, Sara Gariglio, Rodrigo Rocha de Oliveira, Cristina Malegori, Paolo Giordani

**Affiliations:** ^1^ Department of Earth Sciences, Environment and Life (DISTAV) University of Genoa Genova Italy; ^2^ Department of Pharmacy University of Genoa, (DIFAR) Genova Italy; ^3^ Área de Biodiversidad y Conservación Universidad Rey Juan Carlos Mostoles Spain; ^4^ Department of Chemistry and Industrial Chemistry (DCCI) via Dodecaneso Genova Italy; ^5^ Department of Chemical Engineering and Analytical Chemistry Universitat de Barcelona Barcelona Spain

**Keywords:** life‐forms, microclimate, SEM, surface thermal pattern, water content, water loss

## Abstract

Lichens and bryophytes, as poikilohydric and poikilothermic organisms, reach equilibrium with their surroundings. However, non‐vascular epiphytic communities contribute to ecosystem functions, such as water and energy balance, by interacting with the environment through water and heat exchange at the substrate‐atmosphere interface. We hypothesized that variations in water content during dehydration cycles could alter thermal patterns, leading to greater thermal heterogeneity associated with increased life‐form diversity. We captured infrared images of eight bark sample categories representing different epiphytic community compositions. Using structural equation modeling, we analyzed how epiphytic community composition influenced thermal patterns, both directly and indirectly, through water‐related variables. Our findings indicate that foliose lichens and bryophytes exhibited similar water and thermal trends. Both life forms, characterized by higher water content (WC), negatively affected thermal variables. In contrast, crustose lichens had opposing effects on WC and thermal dynamics. From the saturation point, the average WC over five sessions remained above 50% in samples colonized solely by foliose lichens or bryophytes and nearly 80% in those with both. In contrast, samples dominated by crustose lichens had an average WC below 20%. Bark samples with higher bryophyte and foliose lichen cover retained water for longer, whereas those dominated by crustose lichens lost water more rapidly. Regarding temperature, bryophytes and foliose lichens started at approximately 12°C, with mean final temperatures of 13.7°C and 14.4°C, respectively. Crustose lichens had a higher mean initial temperature of 12.5°C and a final temperature of 16.65°C. These differences may be explained by morphological traits, such as the greater hydrophilic properties and higher surface‐to‐volume ratio of bryophytes and foliose lichens compared to the hydrophobic properties and lower surface‐to‐volume ratio of crustose lichens. This study underscores the importance of incorporating non‐vascular epiphytic communities into ecological research aimed at elucidating the regulation of thermal and water dynamics at fine scale levels.

## Graphical abstract

The thermoregulatory mechanisms of lichens and bryophytes at a fine scale can impact the surface thermal pattern. Furthermore, the morphological aspects of non‐vascular epiphytes can drive a different effect in terms of thermal patterns. Our results open the way for new research perspectives on the role of thermal traits within cryptogamic communities by studying the relationship between the functional traits of non‐vascular epiphytes and ecosystem functions, particularly in the context of thermal dynamics.

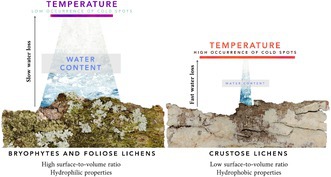

## 1 Introduction

At the ecosystem level, microclimate is of paramount importance as a primary governing factor for many forest functions (de Frenne et al. 2021; Zellweger et al. 2020; Lembrechts et al. 2019). Through the effects of evapotranspiration, redistribution of water precipitation, and absorption of radiation, the understory microclimate is influenced by the vertical and horizontal composition and distribution of the forest canopy (de Frenne et al. 2021; Barbosa et al. 2010; Sprugel et al. 2009; Valladares and Guzmán 2006). The result is a reduction in direct sunlight and wind speed, which modulates air humidity and surface temperature at a fine scale. These effects of forest canopy on microclimatic variations impact the metabolic activity of forest organisms and their contribution to ecosystem functions (Still et al. 2019; Pincebourde et al. 2016). As a result, organisms living below or within tree canopies experience distinct climatic conditions that deviate considerably from the climate outside forests, ultimately modulating their contribution to forest functioning (Chen et al. 1999; Geiger et al. 2009; de Frenne et al. 2019). Among these organisms, non‐vascular epiphytes such as mosses and lichens play a key role by intercepting and retaining water, buffering temperature fluctuations, and modifying humidity levels within and beneath the canopy.

Indeed, as non‐vascular epiphytes, lichens and bryophytes are sensitive to microclimatic conditions, due to their poikilohydric and poikilothermic nature, they cannot actively regulate their own WC and temperature (Proctor and Tuba 2002). These distinctive characteristics make the physiology of lichens and bryophytes highly reliant on the surrounding microclimatic conditions. Their ability to store water and mediate evaporation contributes to the regulation of microclimatic conditions, ultimately influencing the broader forest ecosystem (Ellis and Eaton 2021; Táborská et al. 2020; van Zuijlen et al. 2020). Consequently, air humidity and surface temperature at a fine scale not only determine the response of epiphytic communities but also their contribution to the forest ecosystem functions (Stanton et al. 2023; Schei et al. 2012). Within forests, epiphytic lichens and bryophytes influence moisture partitioning by intercepting precipitation, retaining atmospheric water, and its dynamics. These effects can scale up, contributing to hydrological processes at broader spatial levels, particularly in ecosystems where epiphytes are abundant and contribute significantly to canopy water storage (vanStan II et al. 2020). In turn, this influences canopy water balance and related biogeochemical processes (Porada and Giordani 2021; van Stan and Pypker 2015). Beyond their role in water distribution at larger scales, non‐vascular epiphytic communities also shape microclimatic conditions through water and heat exchange at the substrate‐atmosphere interface (Rice et al. 2018; Kershaw 1983). Their ability to achieve equilibrium with the surrounding environment directly affects the surrounding temperature. For this reason, at a community level, the composition of life‐forms serves as a valuable functional trait, acting as a proxy for the relationships among these organisms with the surrounding microclimate. Rather than comparing mosses and lichens directly, their combined influence on water uptake and release within the forest canopies depends on the diversity of their growth forms and associated functional traits. Crustose, foliose, and fruticose lichens, as well as mosses and liverworts, contribute to water dynamics through their different water retention capacities (Porada et al. 2018). These structural and physiological differences shape critical processes, such as rainfall interception, surface moisture regulation, and heat exchange, particularly at fine spatial scales. Ultimately, it is the community effect—rather than the influence of individual life‐forms—that exerts a greater impact on the surrounding microclimate.

(Porada et al. 2018; Stoy et al. 2012; Lakatos 2011). Indeed, the global change impact on forest ecosystems is even more evident at the fine scale, as non‐vascular epiphytic communities affect the heterogeneity of microhabitats for other threatened organisms (e.g., macro invertebrates; Bokhorst et al. 2015), in terms of microclimatic factors (light, air humidity and temperature), and structural elements (canopy openness, shrub layer and vertical structure of the canopy).

Non‐vascular epiphytic communities offer a promising path to robustly evaluate the relationship between microclimate and ecosystem change. Notably, with the functional traits approach, it has been possible to observe the influence on ecosystem processes exerted by both lichens and bryophytes, particularly those related to water, carbon, and nutrient fluxes (Asplund and Wardle 2017). Research on the density of lichen and bryophyte mats has shown that their thickness influences the soil microclimate. For example, *Cladonia rangiferina*, with a water‐holding capacity of 3.3 L m^−2^, was associated with lower soil temperatures (van Zuijlen et al. 2020). This thermal insulation against temperature extremes and fluctuations is due to the mats' ability to retain water. Also, for bryophytes, independent of mat density, water content was related to thermal conductivity. This may be attributable to the high specific heat capacity and thermal conductivity of water and the different exchange of water and heat, resulting in noticeable humidity peaks on soil between bryophytes and lichens (van Zuijlen et al. 2020; Soudzilovskaia et al. 2013). Therefore, it can be argued that non‐vascular epiphytic communities could also display comparable thermoregulatory processes on tree trunks mediated by their functional traits (Canali et al. 2024; Niittynen et al. 2020; Moore et al. 2019; Pypker et al. 2017; Gersony et al. 2016).

Despite the valuable insights provided by non‐vascular epiphytic communities for understanding the water balance in forest ecosystems (Porada and Giordani 2021; Gauslaa 2014; Porada et al. 2013), the importance of their thermoregulatory effects at a fine scale has been frequently neglected, hampering the comprehensive quantification of the ecosystem functions, such as the forest energy balance facilitated by these organisms. It has been modelled that epiphytic lichens and bryophytes grown on tree trunks lose water rapidly in the morning as the vapor pressure deficit increases. Furthermore, this relationship also showed its greatest effect on the forest energy balance compared to the afternoon affecting the latent heat flux of the forest canopy (Pypker et al. 2017; van Stan and Pypker 2015). Fine scale studies on the influence of non‐vascular epiphytes on thermal characteristics have so far been neglected (Canali et al. 2024). Indeed, there is a lack of data related to understanding the interaction mechanisms of species within a community and the associated water dynamics and thermal patterns. In light of the prevailing climate change circumstances, it is necessary to investigate effects and responses at specific spatial and temporal scales of the considered group of organisms (de Frenne et al. 2021).

The aim of the present paper was to deeply investigate at fine scale how non‐vascular epiphytic communities influence surface temperature both directly on the basis of their morphology and indirectly through different water dynamics within the community. As shown in previous research by Rice et al. (2018) due to different evaporative cooling of shoot apices in bryophytes, different temperature gradients were formed that varied both between species and between canopy structures in relation also to environmental conditions and water status. It is good to think, therefore, that since lichens and bryophytes may have different evaporative cooling due to different water uptake (Porada et al. 2016) this can cause a very heterogeneous thermal pattern within a nonvascular epiphytic community. This goal was achieved by monitoring the surface thermal pattern of samples, characterised by a specific composition of life‐forms, during a dehydration process. Water loss rate (WL) in terms of weight loss between measurement sessions was used as a descriptor of evaporation. For the first time, the impact of community composition was investigated, for understanding the contribution of the different life‐forms (crustose lichens, foliose lichens, and bryophyte) on the surface thermal pattern. As described above, WC affects thermal conductivity (Abu‐Hamdeh 2003). Therefore, it was hypothesised that during a dehydration cycle, changes in water content may lead to variations in thermal patterns. A gradual decrease in WC is expected to lead to an increase in average sample temperature due to the formation of more hot spots. Foliose lichens and bryophytes are hypothesised to retain more water and exhibit slower dehydration dynamics compared to crustose lichens. Consequently, mean temperature is expected to be higher in samples colonised by crustose lichens. Additionally, greater thermal heterogeneity, characterised by a mix of hot and cold spots, is predicted to be associated with more heterogeneous epiphytic communities in terms of life‐forms. The interaction between the life‐forms and the environment through water and heat exchange was analysed by means of structural equation models (SEMs) to define complex relationships patterns between composition, temperature, WC, and WL.

## 2 Materials and Methods

### 2.1 Sampling Design

In a remote area of the Ligurian Apennines (Val Trebbia, Torriglia, località Friciallo in NW‐Italy), 32 bark samples were collected from different chestnut tree trunks ( *Castanea sativa* Mill.). The samples were selected to represent the diverse composition of life‐forms within the epiphytic communities of a sub‐Mediterranean forest. In this context, bryophyte dominant species were *Hypnum cupressiforme* Hedw., *Orthotrichum* sp., and *Frullania dilatata* (L.) Dumort, and they were not distinguished into mosses and liverworts but were considered as a coexistence of them at different extents (Mägdefrau 1982). The dominant species of foliose lichens were *Parmelia sulcata* Taylor, *Flavoparmelia caperata* (L.) Hale, and *Melanelixia glabra* (Schaer.) O. Blanco, A. Crespo, Divakar, Essl., D. Hawksw. and Lumbsch, while the dominant species of crustose lichens were *Phlyctis argena* (Spreng.) Flot., *Lecanora chlarotera* Nyl. subsp. *chlarotera*, and *Pertusaria pertusa* (L.) Tuck. var. *pertusa*. In this regard, bark samples were classified into eight categories based on the composition of epiphytic communities to test the effects of non‐vascular epiphytic colonization on the dynamics of tree surface dehydration and thermal patterns. These eight categories included non‐colonized bark, three epiphytic communities dominated by a single life‐form, and four mixed epiphytic communities: (a) bark poorly colonized (< 5%), (b) bark dominated by crustose lichens (> 95%), (c) bark dominated by foliose lichens (> 95%), (d) bark dominated by bryophytes (> 95%), (e) bark with crustose and foliose lichens, (f) bark with bryophytes and crustose lichens, (g) bark with bryophytes and foliose lichens, and (h) bark with bryophytes, crustose, and foliose lichens.

In the laboratory, to ensure comparability among the samples, the dimension of the 32 bark samples were resized to approximately 5 × 7 cm for subsequent hydric and thermal measurements. To do so, using a hacksaw, the samples were carefully resized and reshaped following the natural crack of the chestnut bark and taking care not to damage the thalli of the non‐vascular epiphytes colonising the bark.

### 2.2 Measurement of the Percentage Cover of Different Life‐Forms

Colour (RGB) images of the samples were captured with a Canon EOS 1100D digital single‐lens reflex camera with 12.2 effective megapixels (4272 × 2848). The RGB images were used for calculating the percentage cover of each life‐form present in the bark samples, including crustose lichens, foliose lichens, bryophytes, and bare bark. For this purpose, the free software ImageJ was used to select regions of interest (ROIs), which can return spatial information about a selected area.

### 2.3 Hydric Traits Measurement

Two functional traits were quantified representing the amount of water present in each sample (i.e., water content, WC; Longinotti et al. 2017) and their dehydration dynamics over time (i.e., water loss, WL; Rundel 1982). Although water uptake and loss are undoubtedly physical processes, these metrics provide insights into the dynamic role that morphology and anatomy play during the hydration and dehydration processes, highlighting their active rather than passive nature. The samples were left for 1 week under laboratory conditions (18°C and 40% relative humidity) and their weight was monitored until dry weight (DW), indicated by weight stabilization for two consecutive days, was reached. Subsequently, the samples were repeatedly water sprayed on the upper surface until full hydration. Once they were completely saturated with water, the maximum wet mass was measured. Afterwards, the samples were gently shaken and blotted three times with dry filter paper for obtaining the wet mass after blotting (WMb) (Longinotti et al. 2017). To monitor the dehydration process, the weight mass (WMt) was measured every 45 min for a total of 15 times. Finally, the water content WC_t_ was calculated for each time as shown in Equation (1), whereas water loss WL was calculated as shown in Equation (2).

(1) $${\mathrm{WC}}_{t}=\left(\frac{{\mathrm{WM}}_{t}-\mathrm{DW}}{\mathrm{DW}}\right)*100$$

(2) $$\mathrm{WL}=\left(\frac{{\mathrm{WC}}_{t}-{\mathrm{WM}}_{b}}{{\mathrm{WM}}_{b}}\right)*100$$

### 2.4 Thermal Pattern Measurement

During the dehydration process, the surface thermal pattern was continuously monitored for each bark sample by capturing infrared images at 45‐min intervals. Surface temperature measurements were collected using a FLIR C5 thermal imaging camera (FLIR Systems Inc.) with a display resolution of 640 × 480 pixels, an IR resolution of 160 × 120 pixels, a spatial resolution (IFOV) of 6.3 mrad/pixel, and a thermal sensitivity of < 70 mK. For this experiment, the emissivity was kept at 0.95 considering that the emissivity for a dry, bare soil is 0.92 while for a green forest it is 0.99 (Table S1; for more information, see Senior et al. 2019).

To extract thermal metrics, thermal pictures were processed previously and transformed into rasters. From these rasters, 49 statistical thermal variables were obtained by using the function *get_stat* in R package *ThermStats* (Senior et al. 2019). These 49 variables comprise patch statistics analysis calculated for hot and cold spots based on the G* variant of the Getis‐Ord local statistic, including metrics such as Area (absolute), Area (proportion), Abundance, Density, Shape Index, Aggregation Index, and Patch Cohesion Index (Getis and Ord 1996). For further details, refer to Senior et al. (2019). After the analysis, the mean, maximum, and minimum temperatures of each bark sample were reported, as well as the spatial thermal pattern indices. To analyse temperature variation over time, we selected mean temperature as a proxy, considering its ecological relevance for epiphytic lichens and bryophytes and avoiding highly correlated variables (Spearman rho < 0.7). In addition, the cold proportion and the hot proportion, which respectively represent the percentage of all pixels that lie within cold or hot spots (Senior et al. 2019), were selected as two thermal diversity indices to describe the thermal patterns.

### 2.5 Statistical Analyses

**FIGURE 1:**
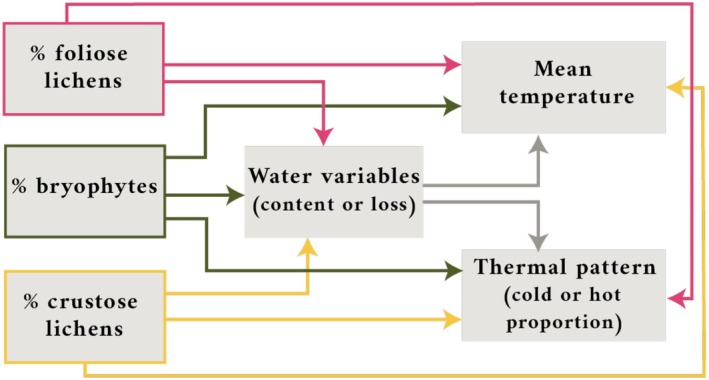
A priori conceptual metamodel illustrating the hypothesised relationships among life‐forms cover, water‐related variables, and thermal‐related variables in non‐vascular epiphytic communities. Specifically, this a priori model tests if the life‐forms directly affect the mean temperature and the cold or hot proportion variables. It also evaluates the indirect effect of the life‐forms through the water variables (water content and water loss) which explain the different aspects of the dynamic cycle of hydration/dehydration. Four independent models were performed relating water content to mean temperature and cold proportion, water content to mean temperature and hot proportion, water loss to mean temperature and cold proportion, and water loss to mean temperature and hot proportion.

To test the relationships between coverage of life‐forms, dehydration dynamics, and thermal patterns, structural equation models (SEMs) were performed using the R package *piecewiseSEM* (Lefcheck 2016). This confirmatory path analysis is a reliable and flexible multivariate method for assessing the directionality of complex connections between variables. Furthermore, this method enables the incorporation of various model structures, accommodates different data distributions, and handles situations with limited sample sizes (Shipley 2009; Grace 2006). A priori conceptual model was proposed and tested to infer how the cover of distinct life‐forms of non‐vascular epiphytes shapes thermal patterns, both directly and indirectly through the dehydration dynamic variables (Figure 1). Specifically, direct paths from non‐vascular epiphytes cover (i.e., percentage of foliose lichens, bryophytes, and crustose lichens) and surface thermal patterns (i.e., mean temperature and hot or cold proportion) were included. Additionally, based on previous studies showing the importance of dehydration dynamics on thermal patterns (Canali et al. 2024), we expected that the diversity of life‐forms may initially affect the dehydration process of bark samples and then these dehydration variables modulate the thermal response. Thus, the indirect paths from epiphytes cover to thermal variables mediated by dehydration variables (i.e., water content or water loss) were also included (Figure 1).

To investigate the effect of epiphytic diversity on hydric and thermal responses, four separate models were fitted, using water content and cold proportion, water content and hot proportion, water loss and cold proportion, and water loss and hot proportion, respectively. The two models containing water content were fitted using linear models, while the two models involving water loss were fitted using linear mixed‐effect models (*lme4* package; Bates et al. 2015) with “time” as a random factor. The fit of each SEM was evaluated using Fisher's C statistic (*p* > 0.05) and based on modification indices, additional paths not initially included were incorporated if the model fit significantly improved. In addition, the residuals of the final models were visually examined to ensure normality and homoscedasticity assumptions. To quantify the proportion of variance explained by the models, two *R* ^2^ were calculated. The marginal *R* ^2^ (*R* ^2^m) represented the proportion of variance explained by the fixed effects alone over the overall variance. The second, the conditional *R* ^2^ (*R* ^2^c), represented the proportion of variance explained by both fixed and random effects over the overall variance (Nakagawa and Schielzeth 2013). We also calculated the variance inflation factor (VIF) to assess the potential impact of multicollinearity on parameter estimates. All analyses were performed using R software version 4.0.3 (Posit team 2025).

The standardized path coefficients of the significant paths were used to measure the magnitude of the direct, indirect, and total effects. Direct effects were represented by the standardized path coefficient when a single significant path connected two variables. Conversely, indirect effects were calculated as the product of the standardized path coefficients in cases involving compound significant paths (Grace 2006). Finally, the total effect of each epiphyte cover was calculated by summing the direct and indirect effects on each thermal variable.

## 3 Results

### 3.1 Water and Temperature Trend Occurring During Dehydration

The WC and its trend during dehydration of the samples is shown in Table S2. The samples that absorbed the most water and released it the slowest over time were the samples colonized by bryophytes and foliose lichens (WC = 108%), followed by the samples colonized only by foliose lichens (WC = 81%) and then bryophytes‐only (WC = 67%). In contrast, samples colonized by crustose lichens only absorbed much less water and dehydrated faster (Figure S1 and Table S2, WC = 22%). This trend is also observed with regard to dehydration dynamics through the water loss rate (WLR, Table S1). With regard to the average temperature of the samples during the dehydration process, it is evident that as the water content decreases, the average temperature of the samples increases (Figure S1 and Table S2). The higher mean temperature at the end of the experiment was observed in crustose lichens (mean T = 16.65°C) while the lower in the samples colonized by foliose lichens and bryophytes (mean T = 13.69°C) All data on the percentage coverage of the different life forms in each sample, information on the environmental conditions during the experiment, and results on water content, water loss, mean temperature, hot and cold spots during the dehydration process are shown in Table S2.

### 3.2 Result From SEM Models

**FIGURE 2:**
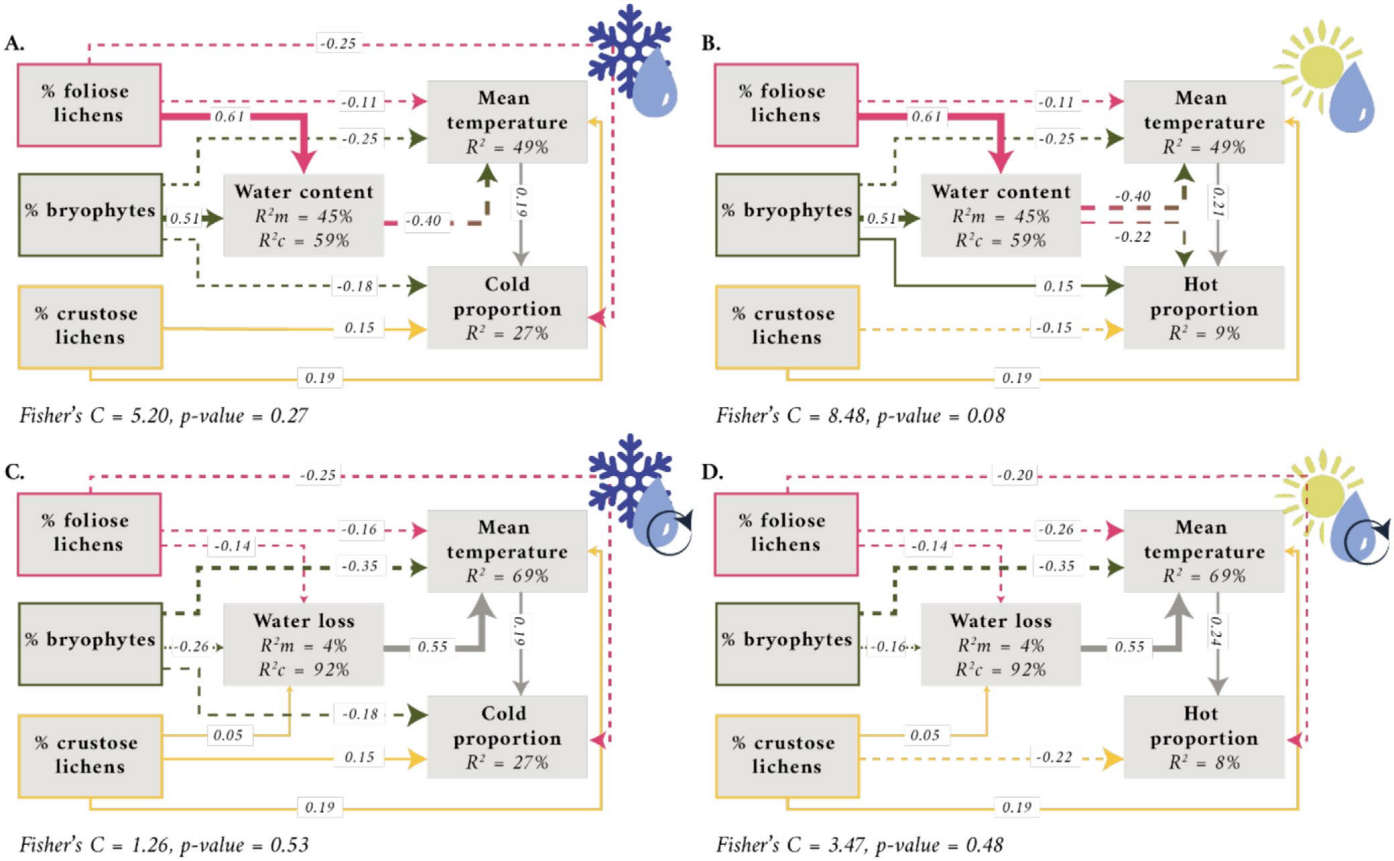
Significant causal paths of the SEM analyses showing the relationships between life‐forms, water‐ and thermal‐related variables. Panels depict the significant paths relating life‐forms' cover with: (A) water content, mean temperature, and cold proportion; (B) water content, mean temperature, and hot proportion; (C) water loss, mean temperature, and cold proportion; and (D) water loss, mean temperature, and hot proportion. In all cases, continuous arrows represent positive relationships (→) and dashed arrows (⇢) represent negative relationships. Arrow width is proportional to the standardised coefficient of the path (indicated by numbers on the lines). The marginal *R* ^2^ (*R* ^2^m) is the proportion of variance explained by the fixed effects relative to the overall variance. The conditional *R* ^2^ (*R* ^2^c) is the proportion of variance explained by both fixed and random effects (random factor = time) relative to the overall variance. Colours refer to different life‐forms depicting foliose lichens in pink, bryophytes in green, and crustose lichens in yellow.

The composition of non‐vascular epiphytic communities influenced water‐related ecological processes, affecting different aspects of the dynamic cycle of hydration/dehydration, such as WC and WL. This influence also extended to the surface thermal spatial patterns, encompassing both cold and hot regions (Figure 2). Specifically, varying levels of life‐forms cover significantly affected the thermal response in the bark samples both directly and indirectly through the influence on dehydration variables (Figure 2). Overall, the composition of the epiphytic communities better explained the water content during the dehydration process (marginal *R* ^2^ = 45% and conditional *R* ^2^ = 59% in WC models; Figure 2A,B) than the dynamic factor of dehydration, which was strongly influenced by time (marginal *R* ^2^ = 4% and conditional *R* ^2^ = 92% in WL models; Figure 2C,D). In all cases, VIF values were consistently less than 2 (i.e., was not detected multicollinearity).

### 3.3 Effect of Life‐Forms on Water Content and Thermal Variables During Dehydration

**FIGURE 3:**
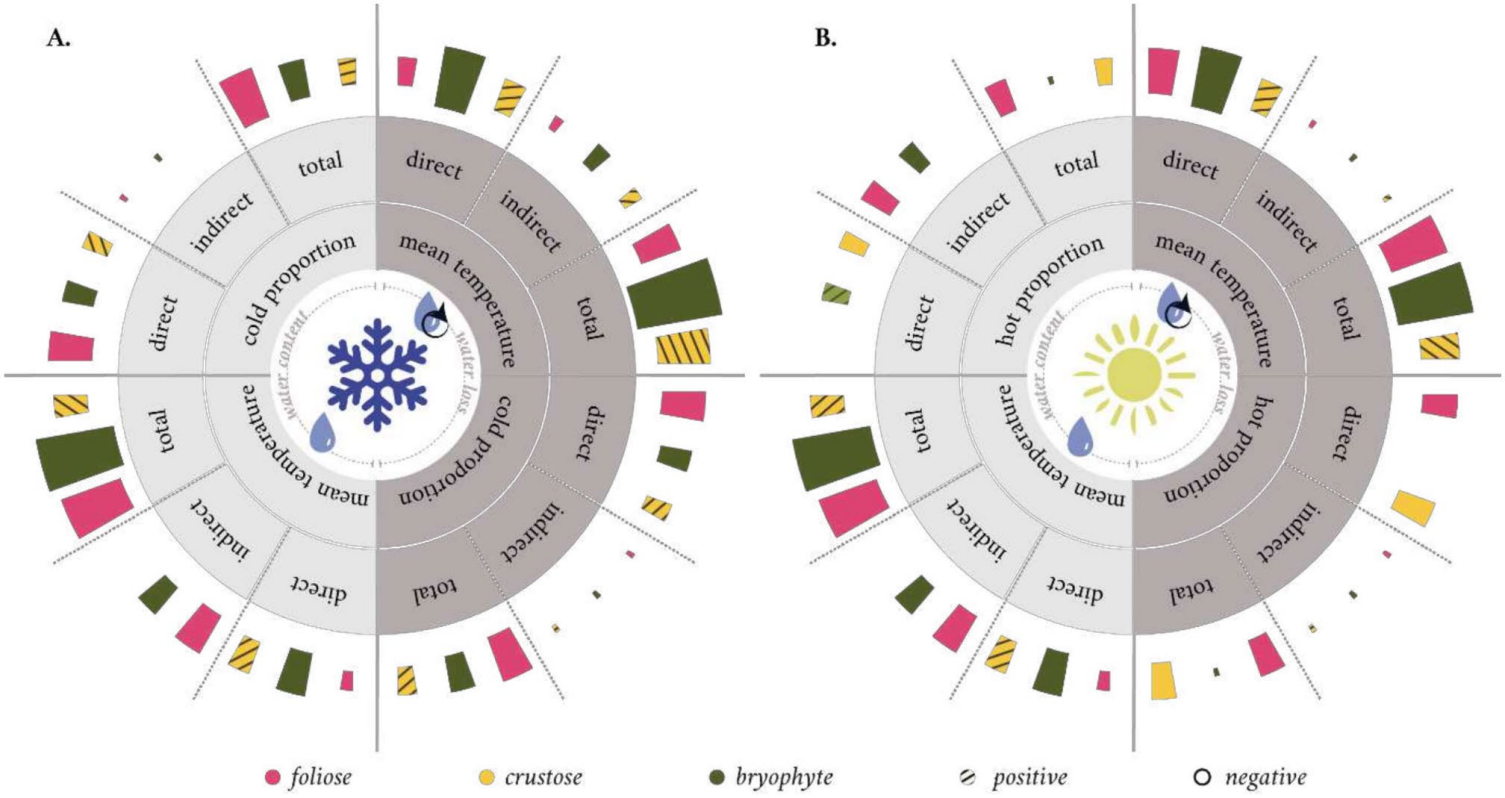
Synthesis of direct, indirect, and total effects of life‐forms on thermal‐related variables obtained by SEM analyses. (A) represents the direct and indirect effect of life‐forms on the mean temperature and cold proportion variable. (B) represents the direct and indirect effect of life‐forms on the mean temperature and hot proportion variable. The standardised coefficient of a path that connects two variables alone is known as the direct effect when there is only one significant path between them (Grace 2006). The standardised path coefficients of the relationship were multiplied to calculate the indirect effects due to the multiple arrows along the compound paths (Grace 2006). By considering all the significant pathways connecting the two variables, the total effect was calculated by adding up both its direct and indirect effects (Grace 2006).

During the dehydration, the WL of samples dominated by foliose lichens and bryophytes was significantly higher than the WC of samples with more crustose lichens (Figure S1 and Table S2). On the other hand, higher WC in samples negatively influenced the thermal variables (Figure 2A,B; Figure S1; Table S2). This indicates that, as the cover of foliose lichens and bryophytes increased within the epiphytic community, there was a rise in bark WC, accompanied by a decrease in mean temperature, as well as in cold and hot proportions. Notwithstanding, both foliose lichens and bryophytes showed similar patterns; foliose lichens showed a stronger positive effect on WC and a more significant role in driving cold and hot proportion variables, whereas bryophytes had a stronger negative effect on mean temperature (Figure 3) and, in contrast to the other models, a positive relationship with hot proportions (Figure 3B). In contrast, crustose lichens did not affect the WC of the bark samples and exerted the opposite effect on thermal variables. This resulted in a higher mean temperature, as well as in higher cold proportions, while direct negative relationships were observed with hot proportions; therefore, as their coverage within the epiphytic community increased (Figures 2A,B and 3).

The influence of life‐forms on thermal variables, both directly and indirectly, has revealed distinct patterns. Bryophytes and crustose lichens consistently exerted a stronger direct effect on mean temperature compared to the indirect effect mediated by WC (Figure 3). In comparison, foliose lichens showed a contrasting trend, with the indirect effect on the mean temperature exceeding the direct effect (Figure 3). Regarding the direct and indirect effect on cold‐hot proportion variables, when first analyzing the cold proportion models, the direct effects of bryophytes and foliose lichens outweighed the indirect effects mediated by WC. An opposite trend was observed for the hot proportion models, in which the indirect effects mediated by WC outweighed the direct effects of bryophytes and foliose lichens. Conversely, the direct effects of crustose lichens in both WC‐mediated models exceeded the indirect effects (Figure 3).

### 3.4 The Varying Effect of Life‐Forms on Water Loss Dynamics and Thermal Response During Dehydration

In terms of dehydration dynamics, both foliose lichens and bryophytes negatively impacted WL, while crustose lichens exerted a positive effect (Figure 2C,D). Although the presence of foliose lichens and bryophytes resulted in a deceleration of WL in the samples, it was observed that a higher cover of bryophytes had a more substantial negative effect on WL compared to foliose lichens (Figure 3). Conversely, crustose lichens had a positive relationship with WL: high coverage of crustose lichens resulted in faster WL within the sample.

As for foliose lichens and bryophytes, they showed a direct negative relationship with mean temperature and cold proportion (Figure 2C). Therefore, with a higher cover of foliose lichens and bryophytes, there is a lower mean temperature and a lower presence of cold spots. In contrast, crustose lichens showed a positive and direct relationship with both mean temperature and cold proportion (Figure 2C). Thus, a greater cover of crustose lichens is associated with a warmer mean temperature and consequently a greater presence of cold spots. Finally, both foliose lichens and bryophytes show a direct negative relationship with mean temperature, while only foliose lichens show a direct negative relationship with the hot proportion (Figure 2D). The presence of foliose lichens therefore correlates with a lower mean temperature (as do bryophytes), while associated with foliose lichens, there is a lower presence of hot spots. As for crustose lichens, they showed a direct positive relationship with mean temperature and a direct negative relationship with the hot proportion (Figure 2D). Therefore, increasing temperature associated with higher coverage of crustose lichens is associated with lower presence of hot spots in the samples. Concerning the influence of life‐forms on thermal‐related variables, even in the case of the water loss models, the direct effect on the mean temperature values of bryophytes and both foliose and crustose lichens was greater than the indirect effect (Figure 3). When evaluating the cold proportion model mediated by WL, the direct effects of bryophytes and foliose lichens outweighed the indirect effects (Figure 3). On the other hand, when analyzing the hot proportion models, the indirect effects of bryophytes, induced by WL, were greater than the direct effects. Conversely, with regard to the effects of crustose lichens in the two models mediated by WL, the direct effects were greater than the indirect effects as in the case of the models mediated by WC (Figure 3).

### 3.5 Overview of the Effects of Life‐Forms on Thermal Variables

From the resulted models, the composition of epiphytic communities better explained mean temperature values (*R* ^2^ = 49 and 69% in WC and WL models, respectively) than cold‐hot proportion variables (*R* ^2^ = 27% and 8%–9% in cold and hot proportion, respectively) during dehydration. Both foliose lichens and bryophytes showed a negative impact on the mean temperature, meaning that a higher cover of these life‐forms decreased the mean surface temperature of the samples. This effect was more pronounced when the samples were dominated by bryophytes (Figures 2A–D and 3). In contrast, crustose lichens had a positive effect on mean temperature, suggesting that their greater cover was associated with the higher mean surface temperature of the bark (Figures 2A–D and 3). Similarly, life‐forms also affected the cold thermal pattern of bark samples, with foliose lichens followed by bryophytes having a lower cold thermal pattern. In contrast, a higher cover of crustose lichens was associated with a greater occurrence of cold spots on the bark surface. Although foliose lichens and bryophytes have consistently exerted similar effects on water dynamics, mean temperature, and cold proportion variables, this trend changes when focusing on the hot thermal model. In this case, both foliose and crustose lichens exerted a direct negative effect on the hot proportion, indicating that a higher coverage of lichens negatively affected the presence of hot spots. In addition, the role of bryophytes in the hot thermal pattern was weak, although they also may decrease the presence of hot spots (Figure 3).

## 4 Discussion

**FIGURE 4:**
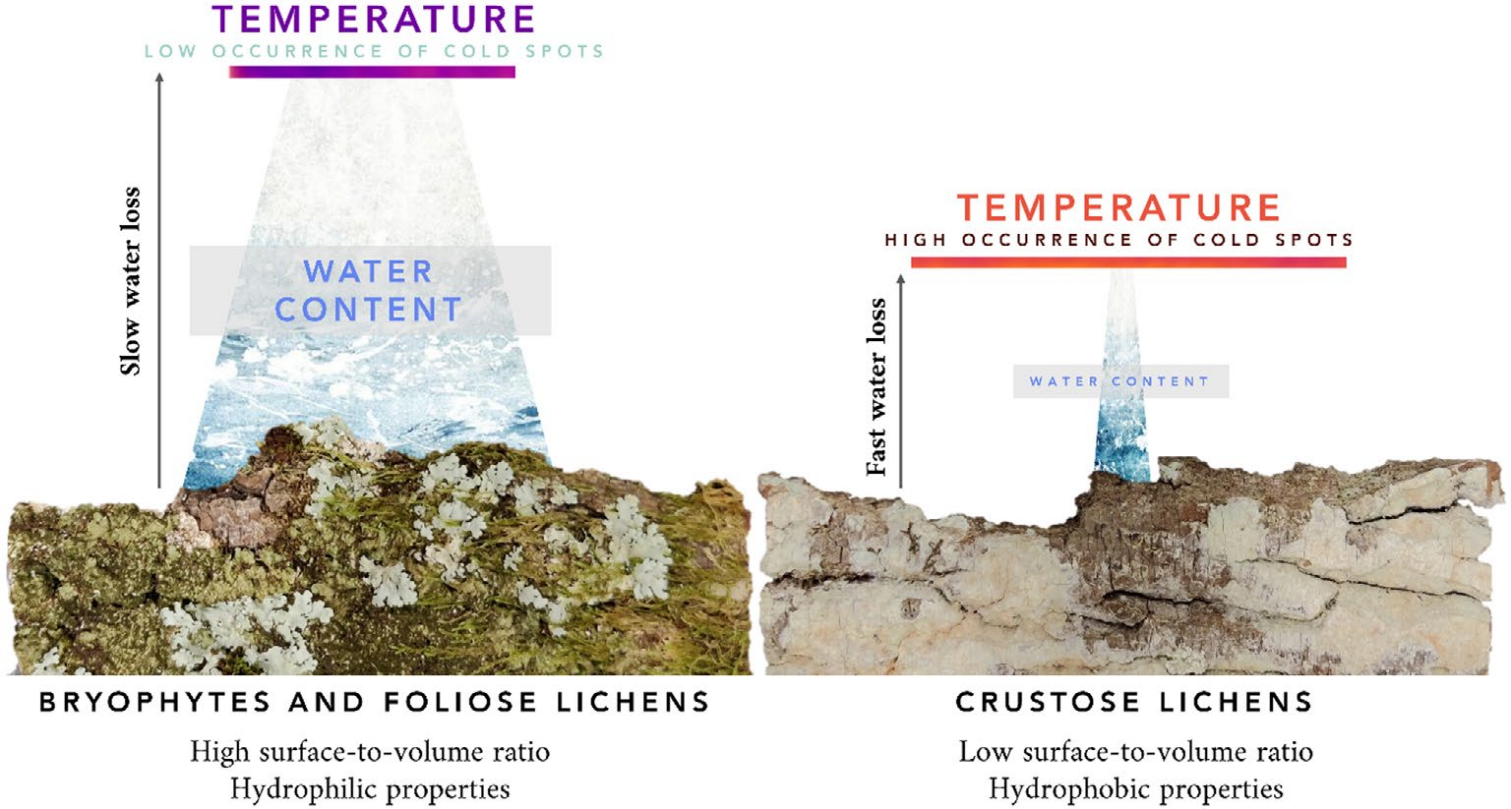
Schematic representation summarizing the main results of the study. Bark samples that display a higher presence of bryophytes and foliose lichens are associated with a longer duration of water retention in contrast to bark samples colonized by crustose lichens, which exhibit a lower water content and faster water loss. In addition, a higher percentage of foliose lichens and bryophytes showed a lower mean temperature; thus, a lower presence of cold proportions (see the temperature bar). Conversely, crustose lichens exhibit a contrasting trend, showing a higher mean temperature and, therefore, show a higher presence of cold spots (see the temperature bar). These trends are driven by the morphological characteristics of the life‐forms.

As noted in a previous work by Canali et al. (2024), at a very fine scale, the functional traits of lichens and bryophytes act as effect traits by influencing surface temperature and moisture. Following this line of inquiry, investigating the fine scale thermal pattern during a dehydration cycle of diverse non‐vascular epiphytic communities provides a valuable tool to clarify the contribution of non‐vascular epiphytes to ecosystem functions. Overall, depending on the percentage of the epiphytic organisms considered, samples may lose water faster or slower based on the poikilohydric and poikilothermic nature of lichens and bryophytes, as non‐vascular epiphytic communities directly interact with the surrounding environment (Kershaw 1983). Consequently, bark samples with a higher coverage of bryophytes and foliose lichens retain a higher amount of water for a longer duration compared to bark samples colonized by crustose lichens, which hold a lower amount of water and lose it at a faster rate (Figure 4). In accordance with our hypotheses, this variation in WC within the epiphytic community indirectly influences the thermal pattern (Figure 4).

### 4.1 Functional Traits Underlying Contrasting Water and Thermal Responses of Foliose Lichens and Bryophytes Versus Crustose Lichens

In terms of water and thermal effects, foliose lichens and bryophytes showed a similar trend. Both life‐forms had significantly higher WC and negatively influenced the thermal variables. However, the relationship between foliose lichens and WC was stronger than that of bryophytes. On the contrary, bryophytes determined a slower rate of WL compared to foliose lichens. These findings may be attributed to the distinct morphology of these life‐forms, where the ‘flatter’ structure of foliose lichens may facilitate heat dissipation, whereas bryophytes, especially the more 3D cushion‐like, dissipate heat more slowly. This could also be related to external water, that is the unabsorbed water on the thallus surface, which constitutes approximately 22% of the total water for foliose lichens (Beckett 1997), while it exhibits a greater variation for bryophytes (Lakatos 2011). The quantity of external water influences the evaporative properties of the thalli but may also directly hinder heat exchange. It is known that lichens and bryophytes have developed morphological adaptations to prevent rapid thallus desiccation, such as low surface‐to‐volume ratio, thick cortices, tomentum, and hair points (Larson 1981). These attributes directly influence the organism's WC and thermoregulatory process, since the growth form is associated with the surface area exposed to interface‐atmosphere exchanges (Rice et al. 2018). Despite their diverse adaptations to environmental conditions, the morphological similarities between foliose lichens and bryophytes outweighed the physiological differences, confirming previous findings (Canali et al. 2024).

On the other hand, crustose lichens showed a contrasting effect to foliose lichens and bryophytes with regard to WC and thermal variables. As their coverage within the epiphytic community increased, the WC decreased while the WL, mean temperature, and spatial thermal pattern increased. In this case, the growth form of crustose lichens differs greatly from foliose lichens and bryophytes, showing a lower surface‐to‐volume ratio, thereby increasing the thallus resistance (Rundel 1982). Furthermore, we assume that this result is mostly influenced by the hydrophobic properties that crustose lichens show. In fact, in order to mitigate the constraints imposed by suprasaturation on photosynthesis, they adopt functional strategies to prevent the formation of water films on their cortex (hydrophobic proteins) and restrict the penetration of free water into the specialised tissues that keep air within the thallus (Lakatos et al. 2006).

Consequently, it appears that the growth form is more related to the presence of hot and cold spots. A more heterogeneous distribution in the surface area and coverage of our samples could be related to the proportion of cold and hot spots and thus the distribution of growth forms in the sample is more influential with the same percentage of surface area. Surely, this denotes a lack of information on this subject and necessary future research.

### 4.2 Perspectives and Plausible Implications of Other Functional Traits

In this work, life‐forms served as a crucial functional trait to investigate water availability and surface thermal patterns at the community level. As discussed in previous sections, the life‐form is a proxy trait that encompasses a number of more complex relationships between the organism and its environment in terms of water, gas, and energy exchange. Nevertheless, the role of additional attributes, both as response and effect functional traits, deserves further investigation to elucidate the intricate relationship among non‐vascular epiphytes, water, and thermal patterns. Despite the similar characteristics of non‐vascular epiphytes within a community, the organisms inhabiting it can vary morphologically and anatomically. These differences emphasize variations in hydration dynamics and define the diversity among non‐vascular species within the community. For example, a high surface‐to‐volume ratio and/or hydrophilic surface is advantageous to absorb more water, while a low surface‐to‐volume ratio and/or hydrophobic properties avoid suprasaturation (Lakatos 2011). If we consider lichens anatomically, they can have a distinctive adapted medulla layer to survive in certain environments (Valladares et al. 1994), as well as bryophytes, which may have different leafy shoots useful in hydration and dehydration dynamics to optimize photosynthetic activity (Proctor et al. 2007; Rice et al. 2001). Thus, emphasizing the anatomical differences, not only between different organisms but also between species, may provide insights into the varying effects of foliose and crustose lichens and bryophytes. Moreover, another important distinction between lichens and bryophytes that may modulate water and thermal patterns is their water absorption strategies. All lichen forms absorb external water by capillary diffusion, while bryophytes employ two main strategies: symplastic conduction (i.e., endohydric) and the exploitation of external capillary water (i.e., ectohydric; Blum 1973). These differing strategies imply distinct hydration and storage dynamics to compensate for saturation, thereby facilitating a photosynthetic activity and impacting thallus desiccation. The adaptive strategies used for water supply may also influence thermoregulatory processes, potentially facilitating or impeding heat exchange.

Another important factor to consider is the intraspecific variation in anatomical and morphological traits among organisms within the same community. Indeed, the composition and physical aggregation between organisms may cause a water reservoir. For example, desiccation stress in bryophytes dense communities may result in higher water content because they have a greater surface capillary space, allowing them to retain water for longer durations compared with individual shoots (Dilks and Proctor 1979).

At a macroscale, the color of lichens and bryophytes, particularly in polar ecosystems, becomes a key trait. For example, light‐colored white thalli reflect light by influencing surface albedo, which in turn influences climate through the fraction of solar radiation that is reflected back into the atmosphere (Aartsma et al. 2020). It is also important to emphasize the role of the environmental factors such as the boundary layer resistance in influencing evaporation. The latter can be highly dependent on wind speed or drying in a context of radiative loading from sunlight. Those factors favor differences within the community. It has also been observed that, at the fine scale of the epiphytic community, the thallus color plays a crucial role in determining the surface thermal pattern (Canali et al. 2024). Despite not considering color as a trait, in this experiment, the major cover of foliose lichens in the samples belonged to the genera *Flavoparmelia* and *Parmotrema*, which are lighter than the dark green color of the genus *Orthotricum* for bryophytes. Notably, previous studies have revealed that the cooling effect derived from lighter colors is generally less than the warming effect from darker thalli (Canali et al. 2024; Gauslaa 1984). Yet, in this work, samples with a higher coverage of foliose lichens and bryophytes show a very similar trend. Therefore, the role of thalli hydric status in modulating the influence of color in thermal response could be the linking key. This aspect might be worthy of further consideration, since the impact of thalli color of epiphytic communities on their surrounding environment has not been quantified yet.

## 5 Conclusions

As observed in this research, the more diverse the community, the more dynamic the water pattern within the community. At this detailed scale, thus, the epiphytic community directly influenced water supply and surface thermal patterns. In turn, at a macroscale, these interactions affect biogeochemical cycles, energy budget, and water balance processes (vanStan II et al. 2020; Pypker et al. 2017; van Stan and Pypker 2015; Cornelissen et al. 2007). Fine scale investigations are thus necessary as they provide additional information, enhancing models aimed at quantifying the ecosystem function of epiphytes at a landscape scale (Porada et al. 2013, 2016, 2018). Remarkably, this study emphasizes the importance of considering the non‐vascular epiphytic community in ecological research, as it can have significant implications for ecosystem dynamics and functioning, such as water balance and energy budget, which in turn, at a microscale, may affect other organisms inhabiting these ecosystems.

## Author Contributions

**Giulia Canali:** conceptualization (equal), data curation (equal), formal analysis (equal), investigation (equal), methodology (equal), writing – original draft (equal). **Pilar Hurtado:** data curation (equal), formal analysis (equal), writing – original draft (equal). **Sara Gariglio:** investigation (equal), writing – review and editing (equal). **Rodrigo Rocha de Oliveira:** methodology (equal), writing – review and editing (equal). **Cristina Malegori:** conceptualization (equal), data curation (equal), methodology (equal), supervision (equal), writing – review and editing (equal). **Paolo Giordani:** conceptualization (equal), data curation (equal), funding acquisition (equal), methodology (equal), project administration (equal), resources (equal), supervision (equal), writing – review and editing (equal).

## Conflicts of Interest

The authors declare no conflicts of interest.

## Supporting information


**Figure S1:** Plot of water content and mean temperature over the time. The image shows the trends in water content, mean temperature and thermal pattern indices over time (*water content*, *WC*; *mean temperature*, *T mean*; *hot proportion*; *cold proportion*). Each trend is subdivided according to its category. Bare bark (woba); bark with bryophyte dominance (bryo), bark with foliose lichens dominance (lifo), bark with crustose lichen dominance (licr); bark with the presence of several groups: bark with bryophytes and crustose lichens (brcr), bark with bryophytes and foliose lichens (brfo), bark with crustose and foliose lichens (crfo), and finally, bark with bryophytes, foliose and crustose lichens (brcf).

**Table S1:** Comparison of different emissivity. This table shows the variations of the maximum (Tmax), minimum (Tmin) and mean (Tmean) temperature as the emissivity varies. In fact, the radiation emitted by an object is a function of its temperature and the amount of radiation emitted depends on its emissivity. The emissivity values given in the table are between 0.92 (for dry, bare ground) and 0.99 (for green forests). In our experiment, the emissivity was kept at 0.95. The categories reported are: bare bark (woba); bark dominated by bryophytes (bryo), bark dominated by foliose lichens (lifo), bark dominated by crustose lichens (licr). Bark with the presence of several groups: bark with bryophytes and crustose lichens (brcr), bark with bryophytes and foliose lichens (brfo), bark with crustose and foliose lichens (crfo), and finally bark with bryophytes, foliose and crustose lichens (brcf).

**Table S2:** Summary of measured variables for each sample category. Category refers to the sample code, while Replicate indicates the number of replicates per category. Time session represents the measurement session. env_RH% and env_T°C denote the air relative humidity and temperature, respectively. % bark, % foliose, % crustose, and % bryophytes represent the cover percentages of bark, foliose lichens, crustose lichens, and bryophytes in each sample. WC% indicates the water content, and WLR% represents the water loss rate of each sample. Mean T°C, hot_prop, and cold_prop are thermal variables extracted using the ThermStats R package, where hot_prop and cold_prop represent the proportion of the sample surface classified as warmer or cooler areas, respectively.

## Data Availability

The data underlying this article are available in Figshare at https://figshare.com/s/8aff04dae049e3079f23.
