# Supplementary figures and images for: How Thermal Patterns Change During Dehydration in Non‐Vascular Epiphytic Communities
Source: Ecol Evol. 2025 Jul 10;15(7):e71756. doi: 10.1002/ece3.71756 (PMC12245620; doi:10.1002/ece3.71756)

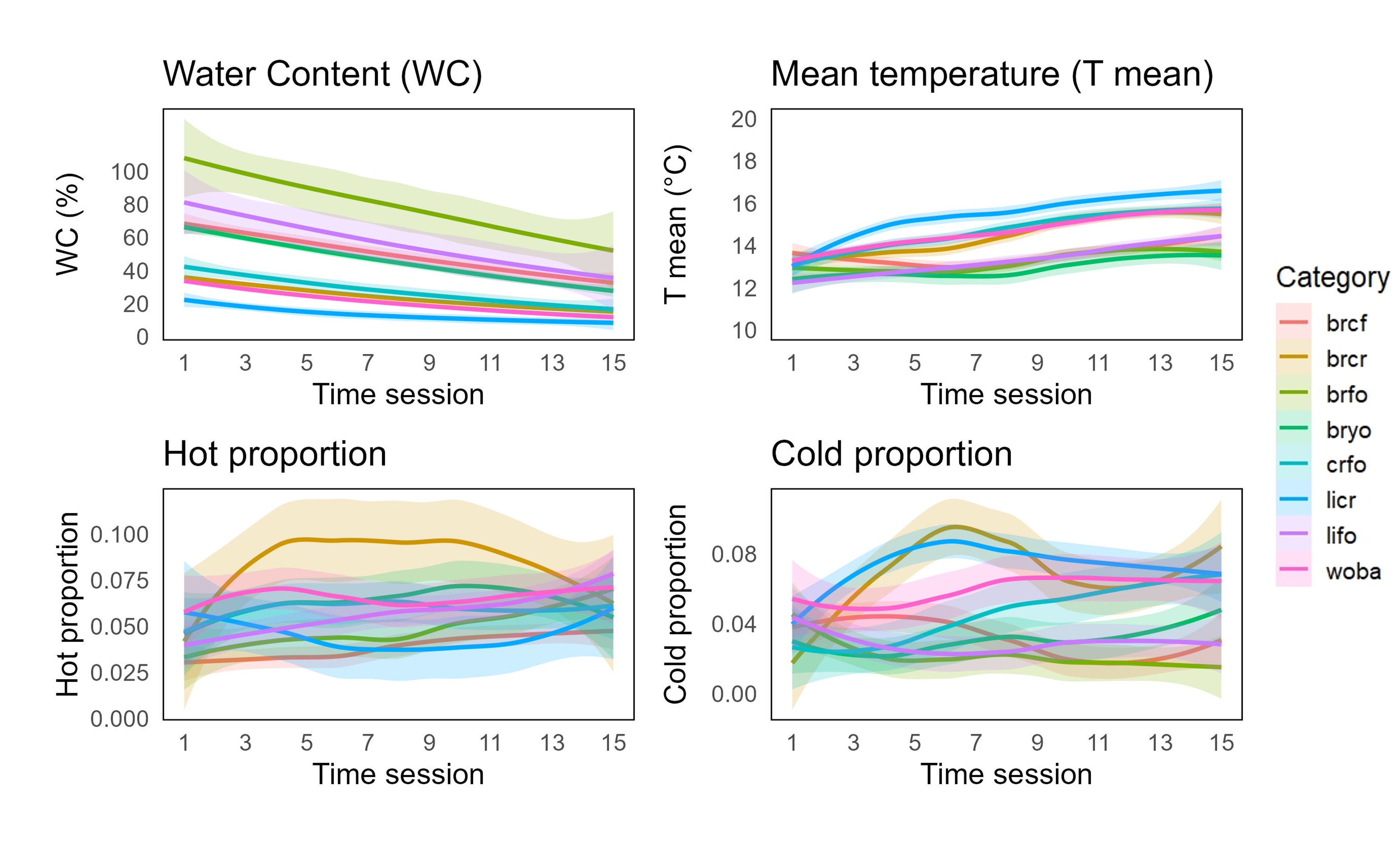

Supplement: Supplementary file 1 — Figure S1: Plot of water content and mean temperature over the time. The image shows the trends in water content, mean temperature and thermal pattern indices over time (water content, WC; mean temperature, T mean; hot proportion; cold proportion). Each trend is subdivided according to its category. Bare bark (woba); bark with bryophyte dominance (bryo), bark with foliose lichens dominance (lifo), bark with crustose lichen dominance (licr); bark with the presence of several groups: bark with bryophytes and crustose lichens (brcr), bark with bryophytes and foliose lichens (brfo), bark with crustose and foliose lichens (crfo), and finally, bark with bryophytes, foliose and crustose lichens (brcf). [file ECE3-15-e71756-s001.jpg]
